# Tobacco dependence and schizophrenia: Tunisian cross-sectional study of 50 cases

**DOI:** 10.1192/j.eurpsy.2022.2151

**Published:** 2022-09-01

**Authors:** M. Jabeur, L. Gassab, I. Anes, A. Ben Haouala, F. Zaafrane, L. Gaha

**Affiliations:** Research laboratory LR 05 ES 10 “Vulnerability to Psychotic Disorders”, Faculty of medicine, University of Monastir, Psychiatry Department, University Hospital Of Monastir, Monastir, Tunisia

**Keywords:** schizophrénia, antipsychotic, dependence, Tobacco-use

## Abstract

**Introduction:**

Tobacco-use is currently one of the major public health problems and is more common among patients with schizophrenia.

**Objectives:**

We aimed in this study to estimate the prevalence of smoking in a population of patients with schizophrenia, to assess tobacco dependence and to identify its correlated factors.

**Methods:**

This is a descriptive and analytical cross-sectional study carried out on 50 outpatients at the Department of Psychiatry (Tunisia) over a period of two months. For the data collection, we used: a general questionnaire on sociodemographic characteristics and tobacco consumption and the Fagerström test for nicotine dependence.

**Results:**

All the patients were male with a mean age of 32.7±7.02 years and 84% of them were tobacco consumers. More than half of the sample were single (68%) and had a primary school level (52%). A professional irregularity and low socio-economic level were found successively in 84% and 78% of cases. Half of the patients (52%) were diagnosed with paranoid schizophrenia and 46% of them were treated by atypical antipsychotics. Cigarette dependence was strong or very strong in 82% according to the Fagerstrom test. A positive correlation was found between strong tobacco dependence on the one hand and low socio-economic level, professional irregularity, smoking in a first-degree relative and treatment with a typical neuroleptic on the other hand.

**Conclusions:**

Our study and data from the literature show that subjects with schizophrenia constitute a population of highly dependent smokers. A smoking cessation assistance program for this vulnerable population is a priority to improve their quality of life.

**Disclosure:**

No significant relationships.

